# Observations on the Evils Resulting from the Premature Application of Artificial Teeth on Plate, with Clasps, Obturators, Palates, &c.

**Published:** 1846-06

**Authors:** John Harris

**Affiliations:** late of George-town, Ky.


### ARTICLE II.

*Observations on the Evils Resulting from the Premature Application of Artificial Teeth on Plate, with Clasps, Obturators, Palates, &c.* By JOHN HARRIS, M. D., D. D. S., late of Georgetown, Ky.

THE resources of art, in supplying defects of the body occasioned by the ravages of disease, or physical or accidental causes, have for a long time been called in requisition, and been attended with various degrees of success, according to the mechanical tact and skill of the surgeon. Much time and talent have been employed in perfecting this department of physical alleviation, especially during the last half century, and rendering it more subservient to the various and complex demands of human affliction.

But the few desultory remarks which I propose to make on the present occasion, will be confined to those cases of deformity, the treatment of which properly belongs to the province of dental surgery, and in doing this, I propose to offer some reasons why the management of such cases should be exclusively confined to practitioners of this branch of the curative art. I allude to defects of the palatine organs, which cannot be remedied by any other than mechanical means, and the application of artificial teeth on plate, &c.

As important as is perfection in the design and execution of all such substitutes, it is equally important that their adoption and use should not be ill advised or premature. The necessity for preparatory treatment, in a large majority of the cases in which essential benefits, if not permanent, might be obtained from their employment, is indispensable, though too frequently it is wholly disregarded or neglected; and when attended to at all, often in a very imperfect and unskilful manner.

In view, then, of the important consideration involved in the treatment of defects of the palatine organs, which can be remedied only by the application of mechanical appliances, it is as necessary for the dentist to be well versed in pathology and therapeutics as it is for the general surgeon. It is alike important to both, and neither can be successful practitioners or safe operators without such knowledge. In supplying the loss of the teeth or any part of the mouth, preparatory treatment is as essential as in the surgery of any other part of the body, and in proportion as such treatment may favor the recuperative powers of the organism, may the operator hope, *cæteris paribus*, to be successful. If, in view of these truths, the *mere* mechanical dentist would be governed by the maxim, "little boats should keep near the shore," he would do much less mischief than he many times does; but this is by no means the case; on the contrary, as a general rule, the greater his ignorance the more he ventures.

A large number of the cases confided to the care and management of the dentist, present a complication of diseases, often involving different and complex tissues, accompanied by more or less general or constitutional derangement. In cases of this sort, and they are of daily occurrence, anatomical, pathological, therapeutical and surgical knowledge is as necessary as mechanical, and therefore they should constitute a part of the elements of the professional character of every dentist.

Such knowledge will reveal to the dentist the nature and causes of the disease or diseases he may be called upon to treat, as well as their proper curative indications. It will also enable him to judge correctly of the necessity and practicability of supplying the loss of the teeth, or of other parts of the maxillary or palatine organs, as well as of the best manner of doing it, and also of the necessity of preparatory treatment, which will often be found to contribute, in an eminent degree, to the usefulness of the artificial appliances he may find it necessary to employ.

There is no branch of manual medicine more difficult than the manipulations of the dentist, and it is seldom that he meets with two cases presenting the same similarity of circumstances. Every new case connects with it some feature or characteristic he may never have noticed before. Hence the difficulty of describing minutely the course most proper to be adopted in each case. This can only be determined by the judgment and experience of the operator.

Preparatory to the application of any dental or palatine substitute, every part of the mouth should be in a healthy condition, and to secure which, it is necessary to extract such of the natural teeth as may be productive of irritation, and to adopt other local and sometimes constitutional treatment. A sufficient time, too, for the parts to undergo all the changes which result in consequence of such treatment, should elapse before the substitute is applied. Months are often required for the completion of these changes, and they frequently give rise to a greater or less change in the position of the teeth contiguous to the vacuity created by the loss of those that have been extracted.

The anomaly thus occasioned demands special consideration on the part of the dentist; it is one that should never be overlooked or lightly appreciated, as the premature substitution of the parts by art would be of no permanent utility, but, in proportion to the subsequent changes in the parts to which the appliance is adapted, would lose its fit, press unequally upon, and become a source of irritation to them. A very trifling undue or unequal pressure, applied even to a sound and firmly articulated tooth, will soon cause it to change its position, and if continued for any considerable length of time, will impair the integrity of its socket, and ultimately destroy the organ. The soft parts too, to which the substitute is adapted, also suffer from its unequal pressure upon them.

Less injurious results could not be expected from a practice so much opposed to the laws of health, although it is frequently resorted to, and pursued to some extent, by men who stand high in the profession. I allude to the application of artificial teeth, mounted on plate, and secured in the mouth with clasps attached to the adjoining natural organs, immediately after the removal of the teeth for which these are substituted. As a general rule, therefore, this practice cannot be regarded as either scientific or judicious, and the practice of applying temporary substitutes for the loss of the natural teeth, when they are to be retained in the mouth by means of clasps, should never be resorted to, except in cases of absolute necessity. If it were necessary, I might mention several cases which have fallen under my own observation, in which sound teeth have been destroyed by the premature and injudicious employment of clasps.

But there are cases in which the application of artificial teeth mounted on plate with clasps, becomes necessary immediately after the removal of the natural teeth, and in such cases the clasps should be made to embrace teeth as far back in the mouth as possible.

The most favorable cases for this description of dental substitutes, and the same may be said with regard to artificial palates, are those in which good, sound natural teeth can be had to serve as a support to them. Therefore, every precaution should be observed and means employed to secure this object, for upon the attainment of which will the permanent usefulness of the mechanism depend.

The following case, which came under my observation a few months since, and if it were necessary I could adduce many other similar examples, will illustrate some of the bad effects liable to result from the premature application of artificial teeth mounted on plate with clasps.

The subject of which was Mr. L., of a little upwards of thirty years of age, scorbutic temperament, teeth originally tolerably good, but from delicate health they had become so much impaired by caries as to render the extraction of the superior incisors, cuspidati and bicuspides necessary. This operation was performed some time in September last; some two or three weeks subsequently to which they were replaced with artificial teeth, mounted on a gold plate, accurately fitted to the parts, and confined by means of clasps applied to the second molars, which at the time were perfectly sound. About four months afterwards, he informed me that he had experienced no inconvenience from the teeth, but on examination of his mouth, I found those portions of the molars embraced by the clasps, and exactly corresponding with them in length and breadth, were very much injured by soft, white caries, which was very sensitive to the touch, while the character of the caries which I at this time observed in several of his other teeth, as well as that which had destroyed those previously extracted, was of a dark brown.

That the retention of the secretions of the mouth between the clasps and the teeth to which they were applied, was the cause of the caries, is very evident, but in a healthy mouth, their action would have been comparatively harmless. At any rate, they would not so readily have acquired such corrosive properties; but even under the most favorable circumstances, artificial teeth thus applied should be removed at least once or twice a day, and the natural organs to which they are clasped be thoroughly cleansed.

In the case in question, the enamel of the teeth was not in the least worn away by the clasps, as would have been the case as its earthy constituents were decomposed, had they been badly constructed and inaccurately fitted.

It is not to be inferred from any thing I have here said, that the mere mechanical dentist is even competent to the management of cases which require no treatment preparatory to the application of a substitute for any of the organs under consideration, for he who is ignorant of the pathology and treatment of disease, must of necessity be equally so with regard to its importance.

As indispensable as it unquestionably is to the practitioner to be able to execute, in the neatest and most accurate manner, a piece of dental mechanism, it is equally necessary to the safe and successful exercise of his professional duties, that he be acquainted with the general principles of medicine.

With regard to the application of artificial obturators and palates, the greater number of the cases which have occurred in my own practice requiring them, have resulted from specific or constitutional causes; and in some of which, when consulted, there was more or less general and local disturbance. The parts were not irritable and inflamed, but in some instances they were in a state of ulceration. In such cases, the restoration of the health of the patient should constitute the first care of the dentist. It would seem almost superfluous to observe, that until this is accomplished, no emergency can justify the application of a plate to the roof of the mouth, yet it is often done. Nor should a plate, whether to serve as an obturator or palate, or support for artificial teeth, ever be fixed in the mouth previous to the full development of the maxilla. The evils resulting from their application under such circumstances are threefold. First, the certain destruction of the natural teeth used as a support to the artificial appliance. Second, the resistance which it offers to the subsequent development of the jaw; and thirdly, the injurious effects arising from the irritation which its presence is sure to occasion.

To illustrate the correctness of the opinion just advanced, I will here give the report of a case furnished some months since, at the solicitation of my brother, Dr. C. A. Harris, and published in the American edition of Fox's Treatise on the Teeth.

Annapolis, *July 28th*, 1845.

Dear Brother,

Your letter of this morning, requesting me to send you, by return of mail, a description of the artificial nose and palate which I made for the young lady of Scott county, Kentucky, is now before me, but received too late to enable me to comply with your request, but I will endeavor to do so by the next succeeding mail.

I very much regret, from the novelty and importance of the case, that you had not allowed me more time, but I will endeavor to do the best I can.

The subject of the affliction requiring the artificial appurtenances referred to, is Miss A. C., now about twenty years of age, of high personal and family respectability.

When but little past infancy, she lost her nose and the central portion of the soft and bony palate, about three-fourths of an inch in length, and three-eighths in width, commencing about five-eighths of an inch in rear of the front teeth, and extending backwards.

I have no knowledge of the cause that led to the affliction, only that it was preceded by inflammation, ulceration, and general constitutional derangement.

When about twelve years of age, she submitted herself to be operated on for an artificial nose, in Cincinnati, Ohio, by Dr. M., the design of which was to supply the defect, by the transfer of integument from the arm, over the deltoid muscle, called the rhynoplastic or telecopeon operation.

To say nothing of the expense, pain and suffering consequent upon the operation, and the jeopardy her life was placed in, as soon as her health would admit, she returned to her home, in a much worse condition than when she left it.

To add to her misfortune, before she left the city, or had fairly recovered from the effects of the first operation, mortified with her now aggravated condition, as a last alternative, she had a nose manufactured of wood, by a Mr. Rostang, resident dentist of the same city, confined in its position by means of spectacles, and an artificial palate, to which the nose was connected by intermediate fixtures, passing through the palatine fissure.

As might have been expected, the subsequent development of the maxilla and other parts soon rendered the whole apparatus useless, though not until it had destroyed the three teeth which had been selected as a support to the palate, by means of as many rough, badly constructed and arranged clasps.

About four years ago, at which time I was consulted in her case, her teeth and relative parts exhibited the following appearance, viz. several of her teeth, besides the three irrecoverably injured by the clasps, were more or less decayed; some of which quite loose; the gums and adjacent soft parts much inflamed, tumefied and spongy, the dental arch and general dimensions of the mouth, (whatever it might ever have been,) was evidently very much collapsed or contracted, say to nearly one-half of the usual size, as was evidenced by the superior cuspidati, now standing parallel to each other, and nearly in contact, between which there had been the usual number of incisors, and of the ordinary dimensions, and which were lost when she was about ten or eleven years of age.

Although the design and plan of the apparatus was a good one for the accomplishment of the purpose for which it was intended, its premature application was unquestionably productive of the worst of consequences, and to which the contraction of the mouth may principally be ascribed.

Her timidity, arising no doubt from the recollection of her previous suffering, gave me no little trouble in obtaining her consent to the course of practice which I recommended; but after explaining what must soon be her condition, she reluctantly yielded her assent, and permitted me the full exercise of my judgment in the management of her case.

The removal of the three teeth previously referred to, was immediately effected, as also all deposites of salivary calculus.

In about eight week, with the usual treatment, the soft parts of her mouth were restored to health. The local maladies of her teeth were next attended to, and in some four weeks, thirteen carious places, after the usual preparatory treatment, were filled with gold foil.

With the mouth thus restored to health, but one question could arise as to the propriety of supplying my patient with another palate, &c.; that was, if the maxillary organs have not attained their full development, the same destructive consequences might be produced, as was from the other palate.

From the circumstance of her advanced age, I did not much fear this would be the case, though, had I been governed entirely by my own inclination, I should have deferred all further proceedings to some more remote period, but in this I was overruled, by not only my fair patient herself, but by all her family connections, and as yet I have not had cause to regret the course I took.

I therefore constructed a palate of fine gold, upon the usual plan, with only two clasps, made broad and heavy, one on the left and one on the right margins of the palate, embracing two of the soundest and most suitable teeth.

To the convex or superior surface of the palate, one end of a piece of gold wire, three-fourths of an inch long, was soldered at a point corresponding with the fissure, from front to rear, and on a line extending between the two teeth embraced by the clasps, describing the centre of action; the wire or upright was then bent forward and upwards, so as to pass through the fissure, and present the upper end parallel with, and at a convenient distance from, the anterior opening of the nares; this place of attachment I found necessary, that no unequal or undue pressure might result from the weight and action of the superincumbent parts upon the palate.

A screw was now cut on a platina wire, for one-half inch, made to fit in a corresponding one made through the upper end of the upright wire, on a level with the external opening of the nose; upon the other, or anterior extremity of the horizontal platina wire, a hook or catch was made as a support to the nose, by means of a gold loop attached to the septum of the artificial nose, the tightness of the nose to be regulated by screwing the horizontal wire in or out, and its position by bending the upright backwards or forwards. The length of the horizontal wire is one inch; the size or strength needs no further description.

It is now about four years since the operation was completed, and thus far I have heard no complaint.

Yours, affectionately,

JOHN HARRIS.

If it were necessary, I might adduce numerous examples, in proof of the pernicious effects resulting from the premature application of artificial appliances in the mouth, but the foregoing will suffice for the present.

### ARTICLE III.

*Case of Fungus of the Inferior Maxillary, successfully Treated.*

